# A New Urinary Disinfectant

**Published:** 1898-12-03

**Authors:** 


### A NEW URINARY DISINFECTANT.

When the urinary passages have become infected so that at the moment of being passed the urine contains bacteria, this condition is generally, no doubt, only a symptom of some diseased condition which itself requires treatment; yet it is often eminently desirable that the urine should, if possible, be rendered aseptic, even though the original malady be not thereby relieved. Often where operative measures are in contemplation it would be worth much to get the urine free from septic organisms before the knife is used, and sometimes, again, when operation is out of the question, the original malady might with comparative comfort be put up with if only the teasing of the bladder by decomposing, and therefore irritating, urine could be put a stop to. Yet this is a thing which, by mere medicine at least, it is most difficult to do. Hitherto the benzoates have held the first place as urinary disinfectants, but Dr. Reynold W. Wilcox,[1] of New York, strongly recommends urotropin for this purpose. This substance, the proper name of which is hexamethylentetramin, is prepared by union of ammonia and formaldehyd in solution, and occurs as colourless crystals, which are readily soluble in water. This drug seems to be excreted by the urine, and possibly in its course is partly decomposed into formaldehyd, for both substances have been found in the urine as early as ten minutes after the administration of urotropin, and as late afterwards as fourteen days. In regard to safety, it appears that 30 grains per day are sufficient, and that from this dose no untoward symptoms have been encountered. Several cases are related from which it would appear that (1) given in the above dose it does no harm; (2) it renders an alkaline urine acid, no matter what the cause may be; (3) it inhibits the development of micro-organisms of ammoniacal cystitis, and thus clears up cloudy urine; and thus (4) is indicated as a preparatory disinfectant in operations upon the urinary tract, and in pyelitis, cystitis, and other conditions tending to the formation of urinary calculi.

[1] New York Med. News, Nov. 12.

